# Evaluation of Emerging Antimicrobials Resistance in Nosocomial Infections Caused by *E. coli*: The Comparison Results of Observed Cases and Compartmental Model

**DOI:** 10.1155/ipid/3134775

**Published:** 2025-01-16

**Authors:** Babak Eshrati, Elaheh Karimzadeh-Soureshjani, Mahshid Nasehi, Leila Janani, Hamid Reza Baradaran, Saeid Bitaraf, Pouria Ahmadi Simab, Sara Mobarak, Sasan Ghorbani Kalkhajeh, Mohammad Kogani

**Affiliations:** ^1^Department of Social and Family Medicine, Iran University of Medical Sciences, Tehran, Iran; ^2^Department of Clinical Sciences, Abadan University of Medical Sciences, Abadan, Iran; ^3^Department of Epidemiology, School of Public Health, Iran University of Medical Sciences, Tehran, Iran; ^4^Biostatistics Department, School of Public Health, Iran University of Medical Sciences, Tehran, Iran; ^5^Department of Community Medicine, School of Medicine, Ahvaz Jundishapur University of Medical Sciences, Ahvaz, Iran; ^6^Department of Clinical Science, Faculty of Veterinary Medicine, Sanandaj Branch, Islamic Azad, University, Sanandaj, Iran; ^7^Department of Infectious Diseases, School of Medicine, Abadan University of Medical Sciences, Abadan, Iran; ^8^Department of Community Medicine, School of Medicine, Abadan University of Medical Sciences, Abadan, Iran; ^9^Department of Biostatistics and Epidemiology, School of Health, Research Center for Environmental Contaminants, Abadan University of Medical Sciences, Abadan, Iran

**Keywords:** compartmental model, *E. coli*, emerging antimicrobial resistance, nosocomial infections

## Abstract

**Background:** In recent years, the global rise of antibiotic-resistant *Escherichia coli* (*E. coli*) has become a significant threat to public health. This study aimed to identify and track outbreaks of antibiotic resistance, specifically among the antibiotics used to treat nosocomial *E. coli* infections.

**Materials and Methods:** This hospital-based study utilized data from a nosocomial infection surveillance system to investigate reported cases of antibiotic resistance. The study analyzed the results of 12,954 antibiogram tests conducted across 57 hospitals in 31 provinces of Iran. The data was divided into two periods: the first and second halves of 2017. Before developing a predictive model for resistant *E. coli* cases, the model's validity was tested using the first half of the year's data. The predicted cases were then compared to the actual observed cases in 2017, with a statistically significant difference indicating an outbreak.

**Findings:** The study found that, in 2017, hospitals in Iran experienced an outbreak of *E. coli* resistant to ampicillin and ceftazidime. This resistance was more prevalent than expected, highlighting the emergence of these drugs as major contributors to nosocomial *E. coli* infections.

**Conclusion:** This study demonstrated the utility of the compartmental model in forecasting outbreaks of antibiotic-resistant *E. coli*. It provides a framework for investigating similar outbreaks in the future, using diverse data sources and methodologies.

## 1. Introduction

A major concern for global public health is the rise of antimicrobial agent resistance, especially in bacteria that cause nosocomial infections [1]. Antimicrobial resistance (AMR) raises the risk of disease, mortality, and healthcare costs [2]. The distribution of bacteria that cause nosocomial infections, particularly those that are resistant to antibiotics, varies over time, between hospitals, and between different areas within the same hospital [3]. AMR among pathogens causing nosocomial infections is a result of the growing number of immunocompromised patients, the use of indwelling devices, and the widespread use of antimicrobial agents in hospital settings, especially in intensive care units (ICUs) [4–6]. The World Health Organization (WHO) has repeatedly emphasized the association of nosocomial infections with AMR and its spread and has identified it as an important public health risk that requires specific interventions [7]. In 2014, the WHO named AMR as a global threat in a report. In this report, the main focus is on antibiotic resistance (ABR). Reasons such as increasing the use of antibiotics in recent decades, widespread misuse of antibiotics in humans and animals involved in food production, lack of new treatment options in the treatment of ABR, and insufficient knowledge about the spread of this type of resistant infection led the focus of this report on ABR [8]. ABR is commonly seen in hospitals due to the high concentration of susceptible patients and the high rate of antibiotic use [9].

As a result of community- and hospital-acquired clinically significant bloodstream infections (BSIs), *Escherichia coli*, often known as *E. coli*, is the most prevalent Gram-negative bacteria that cause a wide range of diseases and is a major cause of death from these infections in people of all ages [10]. Over the past few decades, multidrug-resistant (MDR) *E. coli* has emerged in several nations. The treatment of *E. coli* infections is becoming more and more concerning due to the growing resistance to cephalosporins, particularly the concurrent rise in the prevalence of MDR *E. coli*. The synthesis of plasmid-borne extended-spectrum β-lactamases (ESBLs) is the main mechanism of resistance to β-lactam antibiotics in *E. coli*. Since the initial report in the early 1980s, microorganisms that produce ESBLs have proliferated globally. Transferable plasmids encoding resistance genes often contain ESBL genes, and when commensal or fecal isolates acquire these genes, it results in the development of MDR pathogens [11].

Due to the importance of this issue, at the 68th meeting of the WHO, the Action Plan of AMR was developed in 2015 [12] and launched the Global Antimicrobial Resistance Surveillance System (GLASS) [13]. One of the most important goals of the GLASS program is the timely detection and transmission of Emerging Antimicrobial Resistance (EAR). One of the main ways to identify this “emerging” issue at the national or local level is to recognize deviations from the expected resistance in drug compounds [13]. As a result, it can be said that if the observed cases of ABR are higher than expected, it can indicate an “emerging” situation.

In Iran, increasing microbial resistance has become a concern and a major challenge for the Iranian health system. According to the WHO in the Eastern Mediterranean region, which also includes Iran, there is ample evidence of the emergence of microbial resistance in a wide geographical area. However, there is no way to identify outbreaks caused by these types of infections [14, 15]. Therefore, timely detection and communication of emerging AMR to identify these outbreaks seem necessary. In Iran, according to the GLASS program, the National Nosocomial Infections Surveillance System (NNIS) has been integrated with the AMR program since 2016 and has been implemented in the country's hospitals [16].

In this surveillance system, according to the GLASS program, data related to 7 classes of antibiotics for *E. coli* pathogen are collected: (1) Sulfonamides and Trimethoprim, (2) Fluoroquinolones, (3) Third-generation cephalosporins, (4) Fourth-generation cephalosporins, (5) Carbapenems, (6) Polymyxins, and (7) Penicillins. For each of these classes of antibiotics listed above, the antibiotic agent used for Antibiotic Susceptibility Test (AST) is as follows: (1) Co-trimoxazole (SXT), (2) Ciprofloxacin or Levofloxacin, (3) Ceftriaxone (CEF) or Cefotaxime (CTX) and Ceftazidime, (4) Cefepime, (5) Imipenem and Meropenem, (6) Colistin, and (7) Ampicillin. Therefore, considering the importance of *E. coli*-resistant pathogens in nosocomial infections, it seems possible and necessary to study the differences between the observed cases of this pathogen and its predicted cases to identify outbreaks. In this study, we utilized the Susceptible-Infectious-Resistant (SIR) structure, a type of compartmental model, to estimate expected cases. Each value estimated by this model represents the expected cases, which are then compared with the observed cases using the Standardized Infection Ratio (SIR). If the calculated SIR exceeds 1, it indicates the presence of emerging antibacterial resistance. Therefore, this study aimed to investigate emerging antibacterial resistance using the SIR index (calculated as the ratio of observed cases to expected cases).

## 2. Methods and Materials

### 2.1. Setting

This hospital-based study investigated EAR in nosocomial infections caused by *E. coli* using data from the Nosocomial Infection Care System of Iran. Patients with nosocomial infections in hospitals covered by this care system had their follow-up information recorded, including date of hospitalization, duration of hospitalization, date of infection, type of infection, pathogen causing the infection, patient age, patient sex, type of anti-infection test (Biogram), and its result. In 2017, the system registered 107,670 cases of nosocomial infections, with 15,165 cases related to *E. coli*. This study utilized data from these infections, extracting observed cases of antibiotic-resistant *E. coli* and comparing them with predicted cases determined by a compartment model (separately for each antibiotic) to investigate EAR in this pathogen.

### 2.2. Study Population

The study included patients with nosocomial infections caused by *E. coli* in Iranian hospitals between January and December 2017. All patients admitted to university hospitals located in the centers of 31 provinces (57 hospitals) with nosocomial infections caused by *E. coli* and who underwent antibiogram tests (a total of 12,954 tests) were included. University hospitals were chosen due to their greater cooperation in performing antibiogram tests, the larger sample size available, and their equivalence with public hospitals in accepting a diverse range of patients in different wards. The data from the 12,954 tests were categorized into two parts: part one included data from the first half of the year with 5701 tests, and part two included data from the second half of the year with 7253 tests.

### 2.3. Estimation of Required Parameters and Validation of the Model Used in the Present Study

To assess EAR in this study, the SIR index was employed. The SIR was computed by dividing the observed cases of antibiotic-resistant *E. coli* by the expected cases. If the index exceeded one, it suggested EAR. A 95% confidence interval was established to assess the significance of this index. Inclusion of the number one in the 95% confidence interval indicated nonsignificance, while exclusion suggested statistical significance.

To predict expected cases of antibiotic-resistant *E. coli*, a compartmental model [17] was developed for each of the 11 antibiotics. The population in this model was categorized into compartments Susceptible (S), Infectious (I), and Remove (R). S represented patients with *E. coli* nosocomial infections, I denoted nosocomial infections due to *E. coli* that developed ABR, and R denotes resistant cases of *E. coli* that were discharged from the hospital for any reason (death, treatment). The model predicted the transfer of individuals between these compartments with predetermined coefficients.

**Figure 1:**
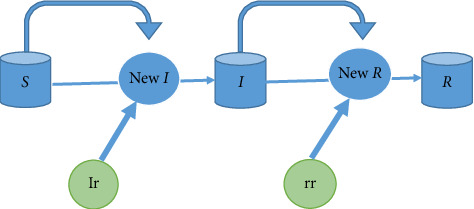
Diagram of developed compartment model. *S*: total number of nosocomial infections caused by *E. coli*; ir: the incidence of resistant *E. coli* per person-day hospitalization; New *I*: the number of cases that have become resistant between *t* and *t* + 1 time; *I*: number of cases of resistant *E. coli* at time *t*; rr: the ratio of resistant *E. coli* discharged from the hospital per day; New *r*: the number of cases of resistant *E. coli* that have been discharged between *t* and *t* + 1 time; *R*: the number of cases of resistant *E. coli* that were discharged from the hospital.

In this study, the probability of transitioning individuals from section S to section I, with the incidence rate (ir), and from section I to section R, with the removal rate (rr), was determined. The “Difference Equations” approach was used to formulate the model, where the transfer of entities between compartments was described in discrete time steps (e.g., days). The “Euler” method, specifically designed for this type of equation, was employed in Berkeley Madonna software version 8.3.23 [18] to set up the model. Graphical diagrams were drawn to set up the models separately for each of the 11 antibiotics (Figure 1). This graphical approach was followed by the formulation of textual equations to represent the model accurately.

The equations (difference equations) generated by the model are shown as follows:

(1) $$\begin{array}{c} S\left(t+dt\right)=S-\text{NewI},\\ I\left(t+dt\right)=I+\text{New}\_I-\text{New}\_r,\\ R\left(t+dt\right)=R+\text{New}\_r. \end{array}$$

*S*(*t* + dt): number of cases of nosocomial infections caused by *E. coli* at *t* + 1 time, *I* (*t* + dt): number of resistant *E. coli* at *t* + 1 time, *R*(*t* + dt): number of resistant *E. coli* removed at *t* + 1 time, T: times used in the model (times between start time and end time), dt: size of time in daily units, in this study, the number one is considered.

### 2.4. Development of Models and Parameter Estimation

During the development of these models, several parameters were required, including INIT S, INIT I, INIT R, ir, rr, start time, and end time. INIT S, INIT I, and INIT R were known parameters with values available at the study's outset. INIT S represented all cases of *E. coli* nosocomial infections with antibiogram tests. INIT I was the number of patients with resistant *E. coli* at the study's beginning, set as “1” in these models. INIT R represented cases of resistant *E. coli* discharged from the hospital at the study's start, considered “zero” in these models. The remaining parameters (ir, rr, start time, and end time) were unknown and needed determination through data examination.

Since this study was the first to investigate EAR in nosocomial infections caused by *E. coli* using the compartmental model, unknown parameters were estimated using data from the first half of the year. When only one data set is available, it can be randomly divided into two parts, with the model developed in one part and its validity assessed in the other. The model was developed in the second half of the year to predict expected cases of resistant *E. coli*. Model validity and performance were evaluated by applying the model to the first half of the year. If necessary, the model was optimized based on the difference between predictions and reality. The total number of predicted cases of *E. coli* resistant to 11 antibiotics was estimated by summing all new cases.

The cross-validation method was employed to evaluate model validity. Before investigating resistant *E. coli* outbreaks in the second half of the year, the model was first applied to assess its validity in the first half of the year. The predicted cases of resistant *E. coli* were compared with the observed cases in the first half of the year. If the predicted and observed cases were similar without a significant difference, it indicated good model performance and validity.

In cases where a significant difference existed, the incidence parameter value was adjusted to minimize the gap between predicted and observed cases of resistant *E. coli*. This adjustment was carried out using the “Optimize” function in the Berkeley Madonna program [18]. The function determined the distance for the ir parameter and performed the model with values within this interval. The output closer to the observed data was selected as the optimized ir. Since the study's available datasets were divided based on time, model validity was considered an intermediate of internal and external validity [19].

## 3. Results and Discussion

The results of the model implemented in the first half of the year are presented in Table 1 to assess the model's validity. This table displays the number of expected cases predicted by the compartment model in the first part of the data for model validation. The “Expected cases” column shows these values, while the last column presents the “Optimized model expected cases.” For CEF, CTX, and SXT antibiotics, the model output (expected cases) significantly differed from the observed cases. By optimizing the parameters, the incidence of these antibiotics was adjusted to be as similar as possible.  Table 2 presents the optimized model developed by antibiotics.  Table 3 presents the number of nosocomial infections caused by *E. coli* in the second part of the data for each antibiotic. The table includes the expected number of cases of resistant *E. coli* predicted by the model, and the SIR index is calculated for each antibiotic.

The findings of this study revealed a significantly higher incidence of ampicillin-resistant *E. coli* among individuals with nosocomial *E. coli* infections compared to the model's predictions. This suggests a significant increase in the incidence of ampicillin-resistant *E. coli*, aligning with studies conducted in Turkey, Iran, and Ireland [20–22]. However, this contrasts with a study in China from 2008 to 2013, which reported a stable incidence of ampicillin-resistant *E. coli* at 82%, indicating a potential occurrence of the outbreak before 2008 [23]. Similarly, a significant increase in ceftazidime resistance was observed among patients with *E. coli* nosocomial infections, indicating a potential outbreak. These results differ from a Chinese study reporting a stable incidence of ceftazidime-resistant *E. coli* at 53% without significant fluctuations [23]. In contrast, observed cases of ceftriaxone-resistant *E. coli* were not significantly different from the model predictions, suggesting a stable incidence during the study period. This aligns with findings in China and Turkey but contrasts with a study in Indonesia conducted between 1985 and 2005, potentially reflecting different study periods and the impact of ceftriaxone use over time. The study also indicated that the observed cases of cefotaxime-resistant *E. coli* did not significantly differ from the model predictions, suggesting a stable incidence during the study period. This aligns with findings in China but contrasts with a study in Indonesia. Similarly, observations of Cefepime-resistant *E. coli* were not significantly different from the model predictions, consistent with a Chinese study reporting a stable incidence at 50% over 6 years. Overall, these results highlight the importance of ongoing surveillance and monitoring of ABR patterns in nosocomial infections. The discrepancies observed between different studies may be attributed to variations in study periods, regional differences, antibiotic usage practices, and infection control measures. Continuous efforts are crucial to understanding and addressing emerging ABR in *E. coli* nosocomial infections.

Since the early 2000s, there has been a rise in *E. coli* resistance to third-generation cephalosporins (3 GCs) across all European nations, largely driven by the dissemination of ESBLs. Jeanvoine et al. [24] reported that a decrease in consumption of 3 GCs and quinolones led to decreasing in resistant *E. coli* isolates. In a 6-year study conducted at a French tertiary hospital, a correlation was observed between decreasing resistance rates to fluoroquinolones and cephalosporins and the reduced use of fluoroquinolones, alongside an increase in the use of antipseudomonal activity penicillin with beta-lactamase inhibitor (AAPBI) [25]. It has been demonstrated that a significant burden of comorbidities, elevated rates of antibiotic use, and recent, frequent hospitalizations are associated with third-generation cephalosporin-resistant *E. coli* isolates [26]. A study in 143 Chinese hospitals in 2014 found a significant link between 3GC-resistant *E. coli* and *K. pneumoniae* and the use of various antibiotics, including *β*-Lactams and cephalosporins [27]. A study in a hospital in Taiwan found that independent risk factors for third-generation cephalosporin-resistant community-onset *E. coli* bacteremia included recent hospitalization, recent antibiotic use, living in a nursing home, having genitourinary disease, and having an intravenous port [28].

Gram-negative strains can acquire resistance through three primary mechanisms: enzymatic resistance leading to antibiotic inactivation, chemical modification of the antibiotic target or use of an alternative target, and alterations in cell permeability or activation of efflux pumps. The first mechanism is identified as the predominant contributing factor among these options [29]. Various ways have been identified as causing *E. coli* to become resistant to ceftazidime, often linked to ESBLs or AmpC *β*-lactamase production alongside active efflux and reduced permeability [30]. Ortiz de la Rosa et al. [31] showed that the ESBL may contribute to resistance against both ceftolozane/tazobactam and ceftazidime/avibactam.

CMY-2 is the predominant plasmid-mediated AmpC β-lactamase expressed by Escherichia coli and various Enterobacterales species, and it is effectively suppressed by Avibactam [32]. Stepwise mutations can increase β-lactamases' resistance to newer β-lactam agents. CMY-185, a CMY-2-like β-lactamase found in *E. coli* from a patient treated with ceftazidime-avibactam, confers high-level resistance to this drug [33, 34]. The CTX-M-15 extended-spectrum β-lactamase was prevalent in a main epidemic *E. coli* strain and many other isolates resistant to cefotaxime and ceftazidime, and the level of resistance may be different among CTX-M producers [35, 36]. The Glu166Arg/Met182Thr mutant of *E. coli* TEM β-lactamase shows a 128-fold increase in resistance to ceftazidime compared to the wild-type enzyme. Experimental data and computer modeling suggest that this mutant confers resistance by a unique covalent-trapping mechanism [37].

The study's results indicate a stable incidence of *E. coli* resistant to levofloxacin over the study period, suggesting no outbreak of levofloxacin-resistant *E. coli*. This stability may imply a positive impact of levofloxacin in treating nosocomial infections caused by *E. coli*. Consistent findings were reported in a study conducted in China, supporting the notion that the incidence of *E. coli* resistance to fluoroquinolones, including levofloxacin, remained constant [38]. In contrast to the model's expectations, a higher incidence of Colistin-resistant *E. coli* was observed among patients with nosocomial *E. coli* infections. However, due to the small sample size and low incidence of Colistin-resistant *E. coli*, these results should be interpreted cautiously. A larger sample size and more antibiogram testing for Colistin are necessary for a comprehensive analysis. Challenges such as the high cost of the Colistin test and hospitals' reluctance to conduct it may hinder a more detailed examination of this resistance. Even a small increase in resistance to Colistin, a last-line antibiotic, poses significant clinical challenges. Colistin is often reserved for treating MDR infections when all other antibiotics fail, and an increase in resistance could limit the treatment options available for critically ill patients. This could lead to higher morbidity and mortality rates, especially in nosocomial infections where patients are already at an elevated risk due to their compromised health. The growing resistance to Colistin would also necessitate the development of alternative treatments and heightened infection control measures to mitigate the impact on patient outcomes. On the other hand, cases of imipenem-resistant *E. coli* among patients with nosocomial infections did not significantly deviate from the model's predictions. The study suggests that the trial performed better than predicted, possibly due to improved health outcomes and the antibiotic's effective action against nosocomial infections. Similarly, the incidence of meropenem-resistant *E. coli* was significantly lower than expected among individuals with nosocomial infections caused by *E. coli*. This suggests that meropenem remains effective against nosocomial infections resulting from *E. coli*. In summary, the study highlights the need for continuous monitoring of ABR patterns, considering the potential variations in resistance across different antibiotics. The differences observed may be influenced by factors such as antibiotic class, regional variations, and healthcare practices. The study provides valuable insights into the dynamics of ABR in nosocomial infections caused by *E. coli*, emphasizing the importance of tailored surveillance and intervention strategies.

The results of this study align with research conducted by Al-Hasan et al. [39], which demonstrated that *E. coli* resistance to levofloxacin and Colistin remained low or stable over time. Furthermore, the findings are consistent with research conducted by Agaba et al. [40], which indicated that the majority of bacteria resistant to other antibiotics are still susceptible to carbapenems. Imipenem was also highlighted as one of the most effective medications for treating nosocomial infections caused by *E. coli* in a study conducted by Ghadiri et al. [41]. These consistent findings across studies suggest that carbapenems, including imipenem, are suitable for treating nosocomial infections caused by *E. coli*.

The study's results on cotrimoxazole-resistant *E. coli* infections also align with Chinese research, where the average resistance to this antibiotic was found to be 60%, and the trend remained constant over the study period [38]. This suggests a consistency in the resistance patterns observed in different regions and reinforces the importance of continuous monitoring and understanding of ABR dynamics. A preprint of the present study has previously been published by Nasehi et al. (2021) [42].

ABR, especially in hospital-acquired infections, remains one of the most pressing challenges in modern medicine. In this study, we utilized a compartment model to predict the incidence of nosocomial infections caused by *E. coli* and examine the patterns of ABR. The results revealed a significant discrepancy between the model's predictions and actual observed resistance, particularly in ampicillin, ceftazidime, and Colistin-resistant strains. This highlights the need for continuous optimization of predictive models to better reflect real-world resistance trends. The findings underscore the critical importance of timely surveillance and monitoring of ABR patterns, particularly in hospital settings, where the emergence of resistant strains can complicate treatment, increase healthcare costs, and lead to higher mortality rates. The study also emphasized the urgent need for enhanced infection control measures and the prudent use of antibiotics to mitigate the escalating threat of resistance. Ultimately, this research demonstrates that ongoing monitoring, combined with effective strategies to curb resistance, is essential for improving clinical outcomes and combating the growing challenge of ABR in nosocomial infections.

## 4. Conclusion

This study highlights the emergence of resistance to ampicillin and ceftazidime in *E. coli*, exceeding expected levels. It underscores the need for careful antibiotic prescribing and the use of AST to guide treatment. The findings can enhance nosocomial infection surveillance and inform policies to combat antibiotic overuse and resistance. These insights offer valuable guidance for clinicians and policymakers to implement effective strategies against ABR. In conclusion, the study provides key insights into the dynamics of ABR in nosocomial *E. coli* infections, emphasizing the importance of vigilant monitoring and prudent antibiotic use in healthcare settings.

## Figures and Tables

**Table 1:** Incidence and optimized incidence of resistant *E. coli* by antibiotic during the year 2017.

Antibiotic	Nosocomial infections	Observed cases	Number of person-days of hospitalization	Incidence rate (95% CI)	Expected cases	*p* value	Optimized incidence	Optimized expected cases
Ampicillin (AMP)	185	150	1926	0.08 (0.07–0.09)	135	N^1^	—	—
Ceftazidime (CAZ)	381	247	3579	0.07 (0.06–0.08)	243	N	—	—
Ceftriaxone (CEF)	498	381	4684	0.08 (0.07–0.09)	343	Y^2^	0.1	385
Cefotaxime (CTX)	364	294	3596	0.08 (0.07–0.09)	251	Y	0.1	281
Cefepime (CFP)	306	218	3124	0.07 (0.06–0.08)	203	N	—	—
Ciprofloxacin (CIP)	714	490	6552	0.07 (0.06–0.08)	455	N	—	—
Levofloxacin (Lvx)	73	48	652	0.07 (0.06–0.1)	48	N	—	—
Colistin (CST)	87	13	934	0.01 (0.008–0.02)	11	N	—	—
Imipenem (IPM)	563	145	5517	0.02 (0.02–0.03)	147	N	—	—
Meropenem (MEM)	319	95	3285	0.03 (0.02–0.04)	114	N	—	—
Cotrimoxazole (SXT)	530	410	4911	0.08 (0.07–0.09)	351	Y	0.1	395

^1^Not significant.

^2^Significant.

**Table 2:** Parameters required to develop the model, by antibiotic.

Antimicrobial agents	ir⁣^∗^	rr⁣^∗∗^	Start time–stop timeᵊ
Ampicillin	0.08	0.1	4–20
Ceftazidime	0.07	0.09	4–18
Ceftriaxone	0.1	0.1	4–18
Cefotaxime	0.1	0.09	4–18
Cefepime	0.07	0.08	4–19
Ciprofloxacin	0.07	0.1	4–18
Levofloxacin	0.07	0.14	3–18
Colistin	0.01	0.06	5–18
Imipenem	0.02	0.08	4–19
Meropenem	0.03	0.09	4–19
Cotrimoxazole	0.1	0.11	4–17

⁣^∗^Incidence rate.

⁣^∗∗^Remove rate per day.

^ᵊ^Period over which the model is run in daily units.

**Table 3:** Calculation of SIR index by antibiotics to evaluate “emerging”.

Antimicrobial agents	Nosocomial infections	Number of resistant's observed	Number of cases expected	SIR	95% CI
Ampicillin	215	192	158	1.2	1.1–1.3
Ceftazidime	568	399	362	1.3	1.02–1.2
Ceftriaxone	575	429	443	1.03	0.96–1.1
Cefotaxime	525	417	405	1.03	1–1.1
Cefepime	338	228	224	1.01	0.90–1.1
Ciprofloxacin	802	537	512	1.05	0.98–1.1
Levofloxacin	122	82	81	1	0.80–1.2
Colistin	151	31	18	1.7	1.02–2.9
Imipenem	761	174	199	0.87	0.73–1.1
Meropenem	389	110	138	0.8	0.65–0.98
Cotrimoxazole	578	433	431	1	0.9–1.1

Abbreviation: SIR, Susceptible (S), Infectious (I), and Remove (R).

## Data Availability

The data are available upon request from the corresponding author.
